# Recent Advances in Multi-Functional Hydrogels

**DOI:** 10.3390/gels12050396

**Published:** 2026-05-03

**Authors:** Liang Chen, Huijie Zhang, Wenshan Ren

**Affiliations:** 1 College of Chemistry and Chemical Engineering, Southwest University, Chongqing 400715, China; chenl0117@swu.edu.cn; 2 Chongqing Key Laboratory of Soft-Matter Material Chemistry and Function Manufacturing, Southwest University, Chongqing 400715, China; 3 College of Bioresources Chemical and Materials Engineering, Shaanxi University of Science & Technology, Xi’an 710021, China; hjzhang@sust.edu.cn

## 1. Introduction

Polymeric hydrogels are an important class of soft materials composed of three-dimensional networks capable of retaining large amounts of water while maintaining structural integrity [1]. Owing to their unique combination of solid-like elasticity and liquid-like transport properties, hydrogels have attracted extensive attention in both fundamental research and practical applications over the past two decades. In particular, multifunctional hydrogels have emerged as highly versatile material platforms because they can integrate mechanical robustness, environmental responsiveness, and application-specific functions within a single material system.

Recent advances in molecular design, network engineering, and materials processing have greatly expanded the performance and functionality of hydrogels. By incorporating dynamic bonds [2], nanofillers [3], interpenetrating or double-network structures [4,5], and topology-optimized architectures [6], researchers have developed hydrogels with enhanced mechanical strength, solvent resistance, conductivity, adhesion, self-healing ability, biocompatibility, adsorption capacity, and controllable responsiveness. These advances have significantly broadened the application potential of hydrogels in biomedicine [7,8,9,10], flexible electronics [11,12], energy storage [13,14,15,16], agriculture [17], environmental remediation [18], soft robotics [19], and other emerging technologies [20,21].

Considering the rapid developments and interdisciplinary significance of multifunctional hydrogels, the *Gels* journal launched this Special Issue, “Recent Advances in Multi-Functional Hydrogels”, to capture the latest advances and foster knowledge exchange in this vibrant field. This special issue comprises six contributions, including four original research articles and two reviews, that collectively showcase the versatility and innovation in contemporary polymer gel research. The selected papers cover diverse aspects of hydrogel design, fabrication, characterization, and application, reflecting the interdisciplinary nature of polymer gel science and its relevance to both scientific discovery and technological development. In the following section, we provide an overview of each contribution, highlighting their research aims, key findings, and significance within the broader context of multifunctional hydrogel materials and technology. Together, these studies illustrate how rational material design and functional integration continue to drive the evolution of hydrogels from conventional water-rich polymer networks toward advanced soft materials with tailored properties and expanded practical applications.

## 2. Overview of the Papers Published in This Special Issue

The first paper “Optimization of the Oxygen Permeability of Non-Silicone Hydrogel Contact Lenses Through Crosslinking Modifications” by Lim et al. investigates a longstanding limitation of non-silicone hydrogel contact lenses, namely their relatively low oxygen permeability, and demonstrates that crosslinking design can be used as an effective strategy to address this problem. The authors synthesized a set of non-silicone hydrogel contact lens materials from N,N-dimethylacrylamide (DMAA) and cyclohexyl methacrylate (CHMA), employing poly(ethylene glycol) dimethacrylate crosslinkers of different ethylene glycol chain lengths and variable concentrations. The authors show that, although all formulations maintained similarly high water contents (about 75.69–80.60%), their oxygen permeability varied markedly from 31.95 to 73.90 Fatt units, with one optimized formulation reaching a level close to that of some silicone hydrogel lenses. At the same time, the materials exhibited generally acceptable optical performance, including high light transmittance and suitable refractive indices. The significance of this study lies in its demonstration that oxygen permeability in non-silicone hydrogels is not only determined by water content alone, but can also be strongly influenced by crosslinker structure and crosslinking level, likely through changes in network architecture and chain entanglement. This finding provides a new design perspective for non-silicone hydrogel contact lens materials and suggests that future research should move beyond simple hydration-based optimization toward more precise network engineering.

The second article, “The Effect of the Conformation Process on the Physicochemical Properties of Carboxymethylcellulose–Starch Hydrogels” by Vedovello et al., explores the preparation of hydrogels made from carboxymethylcellulose (CMC) and starch, crosslinked using citric acid, with glycerol as a plasticizer. This study examines the impact of two different production techniques—casting and extrusion-on the morphology, physicochemical properties, and degree of swelling of the hydrogel. Hydrogels produced by casting formed denser structures without pores, resulting in lower swelling capacities. In contrast, the extrusion method created a more porous structure, leading to enhanced water absorption and swelling, which is advantageous for agricultural applications such as soil moisture retention. This research provides valuable insights into how the processing of biopolymer-based hydrogels can be tailored for specific applications, particularly in improving agricultural water retention and sustainability.

The third paper, “Point-of-Injury Treatment with Hydrogel Containing Dexamethasone Improves Cognitive Function and Reduces Secondary Injury Response After TBI”, by Jones et al. investigates an approach for local, sustained dexamethasone (DX) delivery to treat traumatic brain injury (TBI), focusing on a hydrolytically degradable polyethylene glycol-bis-acryloyloxy acetate (PEG-bis-AA) hydrogel loaded with DX-conjugated high-molecular-weight hyaluronic acid (HA-DXM). The author evaluated the effect of PEG-bis-AA/HA-DXM hydrogel treatment on cognitive function recovery using Morris Water Maze (MWM) test and secondary injury by histological analysis at 14 DPI (chronic injury phase) in a rat moderate-CCI TBI model. The findings demonstrate continued efficacy for PEG-bis-AA/HA-DXM in a moderate-TBI model at 14 DPI, showing improvements in cognitive function and the mitigation of a wide range of secondary injury responses. This work highlights that engineered hydrogels can serve as effective platforms for localized neurotherapeutic delivery, significantly improving functional recovery after TBI.

The fourth paper, “Solvent-Driven Nanostructural Tuning of Lignin/Poly(N,N-dimethylacrylamide) Hydrogels”, by Jiang et al. explores how the composition of solvent influences the nanostructural characteristics and mechanical properties of lignin-based hydrogels. The study demonstrates that, through a solvent exchange process, lignin molecules and poly(N,N-dimethylacrylamide) (PDMA) chains self-assemble to form hydrogels with varied lignin domain sizes and domain distances. A key finding of this study is that the significant role of the Hansen Solubility Parameters (HSPs) of the solvent mixtures in determining the size and arrangement of lignin aggregates within the hydrogel. The research found that balanced solvent mixtures lead to the formation of small lignin domains with high density, enhancing the mechanical strength of the hydrogel. Especially, the formation of large and highly condensed lignin domains enhances the energy dissipation capabilities of the hydrogel, thereby significantly boosting its mechanical properties. The optimized hydrogel exhibited impressive tensile strength (3.2 MPa), Young’s modulus (5.7 MPa), and fracture energy (41.2 kJ m^−2^), outperforming most previously reported lignin-based hydrogels. Additionally, the hydrogels displayed superior adhesive properties and rapid drying characteristics, making them particularly suitable for coating applications. This research underscores the versatility of solvent-driven nanostructural tuning as a strategy for optimizing lignin-based hydrogels, paving the way for their practical use in coatings and other demanding applications.

The Special Issue also features two comprehensive review articles that descript recent progress in specific subfields of multifunctional gels. The first review, “Nano Gel/Hydrogel-Based Components for Battery Technology: An Overview”, by Bhuyan et al. provides the current status and literature background of nanogels and hydrogels in battery technology. This review emphasizes the unique advantages of nanogels, which integrate the swelling behavior and ion-conducting capabilities of hydrogels with the high surface area, tunable nanoscale architecture, and enhanced interfacial properties characteristic of nanomaterials. These characteristics make them promising candidates for a variety of battery components, including gel electrolytes, electrodes, nanogelators, and separator membranes. The authors highlight that nanogel-based batteries could achieve improvements in safety, efficiency, and environmental sustainability, with particular relevance to wearable and flexible electronics. Although nanogel materials have revolutionary advantages, the review points out persisting challenges that need to be addressed. These include scaling up the manufacture of nanogel to kilogram levels while maintaining nanoscale homogeneity, reducing resistance and enhance interfacial compatibility, and lowering production costs. Bhuyan et al. argue that overcoming these challenges will require targeted research focusing on novel material combinations, optimized nanogel production methods, and a deeper understanding of material interactions within battery systems. By clearly articulating both the current capabilities and limitations of nanogel materials, this work establishes a foundation for future research aimed at creating reliable, sustainable, and high-performance nanogel-based battery systems.

The second review, “Hydrogel Systems in Plant Germplasm Cryopreservation: A Comprehensive Review”, by Bobrova et al. provides a systematic and timely overview of hydrogel platforms for plant cryopreservation, with emphasis on how physicochemical properties influence dehydration dynamics, cryoprotectant transport, vitrification stability, and rewarming responses. This work systematically analyzes hydrogel systems used in plant cryopreservation, with particular emphasis on alginate-based platforms; examines how measurable material properties influence key cryogenic processes; assesses performance across explant types and species; and identifies research directions required for the cautious. At the same time, the review discusses recent advances in biomaterials science, including composite polysaccharide blends, protein-based matrices, synthetic polymer networks, macroporous cryogels, and functionalized hybrid hydrogels incorporating surfactants, antioxidants, or nanomaterials. These emerging systems provide greater control over water-binding capacity, pore architecture, solute diffusion, mechanical elasticity, and thermal conductivity, thereby enabling species- and explant-specific optimization of cryoprotective performance. Despite these advances, the review also identifies key challenges that continue to restrict reproducibility and scalability in cryobank applications, including batch-to-batch variability, insufficient standardization of physicochemical parameters such as elastic modulus, mesh size, swelling behavior, and unfrozen water fraction, as well as heterogeneous cryoprotectant diffusion within hydrogel matrices. To address these limitations, Bobrova et al. emphasize the need for mechanistic, materials-driven design strategies and tiered biological validation workflows to reliably link hydrogel properties with cryobiological outcomes. Overall, this review provides an important framework for the rational development of next-generation hydrogel matrices and responsive encapsulation systems, supporting more reliable, scalable, and evidence-based cryopreservation strategies for global plant germplasm conservation.

## 3. Conclusions and Outlook

The contributions to this Special Issue highlight the remarkable versatility and innovation in multifunctional hydrogel research, from structural design to application across biomedicine, agriculture and energy engineering, and optical materials. Several key themes emerge from these contributions. First, innovations in molecular design, network engineering, and material processing enable hydrogels to meet highly specialized functional requirements—from nanogel-based battery components enhancing ionic conductivity and interfacial stability, to lignin/PDMA composites with solvent-tuned nanostructures providing exceptional toughness, and biopolymer gels with tunable porosity for agricultural and environmental applications. Second, the works highlight the critical importance of characterizing and optimizing hydrogel properties, including mechanical strength, rheology, adhesion, swelling behavior, and stimulus responsiveness, demonstrating how multifunctional gels can combine robustness, self-healing, and programmed responsiveness for biomedical, energy, and environmental applications. Third, sustainability and practical implementation are emphasized: for instance, hydrogel encapsulation strategies for plant germplasm conservation showcase scalable, application-ready solutions, while pointing to ongoing challenges such as long-term stability and standardized performance metrics.

Looking forward, multifunctional hydrogels are poised to advance rapidly at the interface of polymer chemistry, supramolecular self-assembly, nanocomposite integration, bioinspired hierarchical design, and additive manufacturing. Future research will focus on achieving multiscale structure–property–function optimization, programmable responsiveness, robust mechanical performance, long-term stability, and scalability for diverse applications in healthcare, environmental protection, sustainable agriculture, energy technologies, and electronics. By bridging fundamental principles with practical applications, these efforts are expected to accelerate the translation of hydrogel innovations from laboratory research to industrial and clinical deployment, further establishing multifunctional hydrogels as a cornerstone of next-generation advanced materials.

In conclusion, this Special Issue emphasizes the synergistic relationship between molecular design, hierarchical architecture, and responsive functionalities in addressing complex scientific, technological, and societal challenges. By highlighting innovative strategies, practical applications, and current limitations, this Special Issue offers valuable theoretical and technical guidance for interdisciplinary researchers, and charts a path for accelerating the translation of hydrogel innovations from laboratory research to industrial, clinical, and environmental implementation.
